# Analyzing the varied impact of COVID-19 on stock markets: A comparative study of low- and high-infection-rate countries

**DOI:** 10.1371/journal.pone.0296673

**Published:** 2024-01-11

**Authors:** Sharon Teitler Regev, Tchai Tavor

**Affiliations:** Department of Economics and Management, The Max Stern Yezreel Valley College, Yezreel Valley, Israel; Universiti Malaysia Sabah, MALAYSIA

## Abstract

The global health crisis initiated by the COVID-19 pandemic triggered unparalleled economic upheavals. In this comprehensive study of 16 countries categorized by their infection rates, we scrutinize the impact of a range of variables on stock market indices and calculate four critical ratios derived from those variables. Our regression analyses reveal striking differences in how the variables influenced stock indices in countries with low and high infection rates. Notably, in countries with low infection rates, all variables exhibited significant effects on stock returns. An increase in infection numbers and fatalities correlated with greater stock market declines, underscoring the market’s sensitivity to the health and economic risks posed by the pandemic. Recovery and testing rates also displayed positive associations with stock returns, reflecting investor optimism concerning potential recovery scenarios. Conversely, nations grappling with high infection rates experienced notably weaker effects from these variables. Although fatalities had a negative impact on stock indices, other factors, including recoveries, infections, and testing rates, did not result in significant effects. This suggests the likelihood that markets in high-infection countries had likely factored pandemic conditions into their pricing, thereby reducing the immediate impact of these metrics on stock returns. Our findings underscore the intricacies of the COVID-19 pandemic’s impact on stock markets and highlight the importance of tailored strategies and policies for distinct country categories. This study offers valuable insights for policymakers and investors navigating financial markets during global health crises and preparing for future epidemics.

## Introduction

The year 2020 will be remembered as the year of a public health crisis caused by the COVID-19 virus, which led to immense economic disruptions throughout the world, some unprecedented in history. The health emergency and the extreme measures implemented to slow the spread of the virus generated economic crises worldwide, the full extent and outcome of which remain to be fully ascertained and understood. This event was unique, with the virus spreading swiftly in nearly every country in the world. The economic shocks, first appearing in December 2019, initially affected specific sectors, such as hospitality and culture, before extending to encompass entire markets. Schools closed, employees faced layoffs, and supply chains were disrupted, leading to declines in consumption and investment. Notably, the impact of COVID-19 on the U.S. stock market more closely resembles the repercussions of the major economic crises in 2008, 1987, and 1929 than it does the consequences of other outbreaks of infectious disease [1].

Motivated by these extraordinary circumstances, our study investigates how COVID-19 affected capital markets in 16 countries. We classify these countries into two distinct groups based on their infection rates: one characterized as having a low infection rate and the other as having a high infection rate. We then gather two types of data: pandemic impact metrics, consisting of numerical data, and pandemic response metrics, comprising categorical data. Utilizing a comprehensive methodological approach, with ten variables and four calculated metrics, this research seeks to offer valuable insights for capital market participants and policymakers.

Our study was conducted during the initial wave of COVID-19 from January 2020 to June 30, 2020, which marks the onset of the pandemic. This is a crucial period for examining the measures taken by different countries and their economic effects in order to better prepare for future health crises [2]. Focusing on this early, uncertain phase, our research pre-dates the development of public health strategies and the introduction of vaccines and treatments that subsequently reduced financial market volatility. Our scrutiny of this period offers vital insights into the economic ramifications of health crises that will inform future preparedness, policy development, and investment strategies within capital markets.

This study’s unique contributions are multifold. It diverges from the norm by encompassing a broad international perspective, analyzing 16 countries categorized into low and high infection rates. Furthermore, it adopts a comprehensive dataset comprising ten variables, classifying them into pandemic impact metrics and pandemic response metrics. This study is also unique in its inclusive use of diverse data sources, including previously omitted categorical data on public behavior and government responses, which facilitates a comprehensive analysis of the epidemic’s impact. Notably, it offers a balanced assessment by investigating both positive and negative indicators of the pandemic. Its temporal focus on the initial outbreak wave of COVID-19, a period marked by significant effects on capital markets, distinguishes it from studies concentrating on later phases or shorter timeframes. Thus, this research enriches the existing literature by providing a nuanced and insightful understanding of how the COVID-19 pandemic affected capital markets.

The findings reveal that in countries with low infection rates, all variables significantly affected stock index returns. In countries with high infection rates, the effects were weaker and were observed for fewer variables. Generally, higher numbers of deaths and infections correlated with larger stock market declines, whereas higher rates of recovery and testing had a positive impact on stock markets. Government-imposed restrictions and public unresponsiveness resulted in declining market indices, whereas proactive measures led to increased returns, as did the easing of workplace and educational restrictions. Notably, the variable for VIP (very important personnel; e.g., political leaders, medical personnel, and security personnel) infections was associated with a decrease in returns.

## Literature review

### Analyzing the influence of health crises and disasters on stock markets

This literature review explores the findings of a range of studies on the impact of diseases and pandemics on stock markets. The effects of natural disasters, health crises, and even acts of terrorism on economic activities have garnered extensive academic attention, particularly regarding the intensification of such events. Tavor and Teitler-Regev [3] conducted a comprehensive study exploring the impact of different event types on stock markets. Their research revealed that among these events, natural disasters were the most damaging to the economy, while terrorism and artificial disasters had the lowest economic impact. Chen’s [4] study in Taiwan revealed that extreme incidents, including earthquakes, the 9/11 terrorist attacks, and the 2003 severe acute respiratory syndrome (SARS) outbreak, significantly affected hotel sales, but had a comparatively mild impact on the stock prices of hotel companies. Chopra and Mehta [5] expanded the scope of research by examining the effects on Asian stock markets of major crises, including the Asian financial crisis, the U.S. Supreme Court crisis, the Eurozone debt crisis, and the COVID-19 pandemic. Their investigation identified the U.S. Supreme Court crisis as the most damaging among these events.Wu et al. [6] conducted an empirical investigation to assess the impact of the subprime mortgage crisis, the European debt crisis, and the COVID-19 pandemic on 36 stock markets. The study sought to identify abnormal changes in these markets during periods of financial crises.

In the context of disease, Donadelli et al. [7] explored the role of media coverage and World Health Organization (WHO) alerts in shaping investor attitudes and influencing pharmaceutical stock prices in the United States. Their findings indicated that media coverage of infectious diseases and WHO alerts had a positive influence on investor sentiment on Wall Street. Similarly, in a study that spanned the United States, China, the United Kingdom, and Japan, Bai et al. [8] examined the impact of infectious disease pandemics on stock market volatility from 2005 to 2020 in. They found a substantial positive influence on the longer-term volatility of these markets, with effects persisting for up to 24 months. Notably, the actions taken by individual countries in response to pandemics produced varied outcomes on their respective stock markets. Schell et al. [9] investigated the influence on stock markets of six WHO announcements from 2008 to 2020, finding that, with the exception of COVID-19, diseases did not exert significant effects on stock markets within a 30-day window.

Some research has concentrated on specific diseases. Pendell and Cho [10] focused on the outbreaks of foot, and mouth disease in Korea between 2000 and 2010, finding a range of stock market reactions, including both anticipated and unexpected responses, with cumulative abnormal returns (CAR) extending over several days. Chen et al. [11] centered their study on the impact of the SARS outbreak in Taiwan, employing an event-study approach to assess the outbreak’s effect on hotel stock prices. They found a sharp decline in earnings and stock prices for seven publicly traded hotel companies during the outbreak that resulted in significantly negative cumulative mean abnormal returns. Additionally, Ali et al. [12] examined what they found to be the dramatic impact of the SARS outbreak on the Malaysian stock market, and Nippani and Washer [13] explored the effects of SARS on stock markets in a range of countries, concluding that only China and Vietnam exhibited significant impacts. Ichev and Marinč’s [14] investigation of the effect of the Ebola outbreak on the U.S. stock market found that the greatest impact was on the stocks of companies exposed to West African countries or to events in these countries.

### The global impact of COVID-19 on stock markets

The COVID-19 pandemic has sparked an extensive body of research into its far-reaching effects on global stock markets and economies. For example, Huang [15] observed that in the United States, the implementation of social distancing policies contributed to a mitigation of the spread of COVID-19. Conversely, Zhang et al. [16] conducted a study examining the impact of the lockdown in Shanghai on sentiments expressed in social media. Their findings revealed a generation of negative sentiment among Shanghai residents and a concomitant generation of positive sentiment among individuals residing outside Shanghai. Researchers have explored the pandemic’s impact at both the national and international levels, focusing on diverse metrics and variables. Their investigations have yielded numerous insights into the intricate relationship between health crises and financial markets. Several studies have adopted a single-country approach, investigating how the stock markets of specific countries responded to the pandemic. For example, Hatmanu and Cautisanu’s [17] research offers valuable insights into Romania’s stock market dynamics during the crisis. Liu et al. [18] focused on China, providing a detailed analysis of the pandemic’s effects on the stock market there. Likewise, Bao and Huang [19] scrutinized the impact of the COVID-19 pandemic on both conventional bank loans and Financial Technology (FinTech) loans within the Chinese financial landscape. Their findings revealed a discernible escalation in the rate of FinTech loans amid the pandemic, whereas bank loans exhibited no statistically significant alteration. Furthermore, Li et al. [20] conducted an empirical inquiry into the repercussions of lockdown measures and governmental subsidies, elucidating their influence on rural enterprises in China during the initial phases of the COVID-19 crisis. Their investigation indicated that the effects were contingent upon demographic variables. Subsequently, in a distinct study, Li et al. [21] highlighted economic losses in rural China attributed to logistical disruptions stemming from lockdowns. Finally, Zhou [22] conducted an examination into managerial beliefs and prevailing business conditions both preceding and during the COVID-19 pandemic. Their analysis revealed a transient alteration in managerial beliefs during the pandemic.

In addition to single-country analyses, researchers have conducted comparative studies to identify disparities in market reactions across countries. Tetteh et al. [23] conducted a comparative study of Ghana and Botswana, which yielded a nuanced understanding of the differing impacts on these two African countries. Meanwhile, Ganie et al. [24] expanded the scope of their analysis comparing stock market reactions in the United States, Brazil, India, Russia, Mexico, and Spain, thus providing a broader perspective on the pandemic’s financial ramifications. Global comparative studies have also been instrumental in shedding light on market dynamics across countries. Ali et al. [25] conducted a comprehensive assessment of the correlation between the worldwide spread of COVID-19 and its impact on financial markets, revealing notable differences between global financial markets and the Chinese market. Singh and Shaik’s [26] examination of the effects of WHO announcements on various sectors found significant negative impacts on all markets, albeit with distinctions between developed and emerging economies.

In the context of the United States, researchers have closely scrutinized the country’s unique stock market characteristics and their interactions with the pandemic. Baker et al. [1] reported an unusual number of markets jumps during a specific period, for which they offered multiple explanations, including the severity of the disease and the policy responses to the pandemic. Chowdhury and Abedin [27] employed an event-study approach to scrutinize the U.S. stock market’s response to confirmed COVID-19 cases and death tolls. Similarly, Chowdhury et al. [28] conducted a comprehensive analysis from January 2020 to April 30 2022, that highlighted the market’s sensitivity to the number of deaths and the positive market response to government financial aid announcements.

Market responses worldwide have been evaluated across various timeframes, from the short term to more extended periods studies adopting this longitudinal approach have provided insights into the nuanced effects of COVID-19 on stock markets and the factors influencing these outcomes, underscoring the intricate relationship between the pandemic and the financial markets as well as the importance of tailoring strategies to navigate these complex interactions [29–32]. Additionally, the media’s role during the pandemic has also drawn attention, with researchers investigating its impact on investment sentiment and equity market volatility. In this realm, Haroon and Rizvi [33] discerned a substantial contribution by the media to investment uncertainty, while Teitler-Regev and Tavor [34] found differential effects between the market situation and that of the global landscape in a range of variables related to COVID-19.

## Hypotheses and theoretical framework

Drawing from the empirical evidence presented above, we posit the following:

Hypothesis 1 (H_1_): Pandemic impact metric variables exhibit different effects on stock indices when applied to countries with low versus high infection rates.
Hypothesis 2 (H_2_): Calculated metrics exhibit different effects on stock indices when applied to countries with low versus high infection rates.

These hypotheses are based on a robust theoretical framework and are substantiated by empirical evidence garnered from an extensive review of the existing literature. As shown in the work of Tavor and Teitler-Regev [3] and Chopra and Mehta [5], among others, the type and severity of events exert differential influences on financial markets. This underscores the importance of considering various metrics to comprehend the unique repercussions of health crises, particularly within the context of countries marked by varying infection rates. Moreover, the pivotal roles of information and media coverage in shaping investor sentiment during pandemics, as emphasized by Donadelli et al. [7] and Bai et al. [8], highlight the influence of diverse pandemic impact metric variables as critical determinants of investor behavior and, consequently, stock market dynamics.

The diverse stock market responses to various pandemics, as clarified by Schell et al. [9], indicate that not all pandemics have identical effects on financial markets, especially when disparities in infection rates among countries are taken into account. Accordingly, H_1_ postulates that pandemic impact metric variables, including the number of infections, deaths, recoveries, and tests conducted, will result in distinct consequences for the stock indices of countries according to whether their infection rates are high or low. Hypothesis 2 posits that the calculated metrics for deaths per infection (DPI), recoveries per infection (RPI), tests per infection (TPI), and tests per death (TPD) will have diverse effects on the stock indices of different countries, particularly when applied to countries with low and high infection rates. Thus, our hypotheses take into account the nuanced and multi-dimensional nature of stock market responses to pandemics, recognizing that each metric makes a distinct contribution to the overall market impact.

## Material and methods

### Data

Our research explores the impact of COVID-19 on the capital markets in 16 countries, covering the daily data from January 2 to June 30, 2020. The countries are divided into two distinct categories: those with high infection rates (the United Kingdom, Italy, Spain, Sweden, France, Germany, the United States, and Brazil) and those with lower infection rates (New Zealand, Austria, Slovenia, Argentina, China, Taiwan, Singapore, and Israel). The selection of these countries was based on the prevalence of COVID-19 infections, encompassing both significantly high and low incidence rates. Furthermore, the inclusion criteria involved a requisite consistency in the reporting of epidemic data by these countries. Thorough documentation of the data sources utilized in the computation of these variables is accessible in Appendices A and B in S1 File.

Table 1 describes the variables collected for regression analysis. In Panel A, the variables are divided into two primary groups: pandemic impact metrics, which consist of numerical data; and pandemic response metrics, which encompass categorical data. The pandemic impact metrics comprise four quantitative variables representing the number of infections, deaths, recoveries, and tests conducted. The pandemic response metrics comprise six categorical variables representing governmental and public responses to the epidemic: restrictions on movement, public behavior, VIP, positive government measures for dealing with the pandemic, restrictions in the education system, and workplace restrictions. Panel B provides a detailed description of four calculated metrics to enhance the robustness and reliability of the regression results obtained from the initial variables. These metrics, DPI, RPI, TPI, and TPD, contribute to a more comprehensive data evaluation.

**Table 1:** Overview of the explanatory variables.

Panel A: Collected variables		
Variable	Details	Values
Pandemic impact metrics variables		
Infections	Numbers of people infected by COVID-19	Exact value collected
Death	Numbers of COVID-19 fatalities	Exact value collected
Recovered	Numbers of people who recovered from COVID- 19	Exact value collected
Tests	Numbers of tests for COVID-19 performed in the country	Exact value collected
Pandemic impact metrics variables		
Restrictions	The level of restrictions in the country including closures and travel bans, restrictions on citizens’ movements, limitations on tourists coming into the country, lockdowns, and isolation measures	-1 = New restriction applies
		0 = Unchanged
		1 = Restrictions are removed
Public behavior	Public responsiveness to government instructions	-1 = Disobeying the government’s instructions
		0 = Unchanged
		1 = Obeying the government’s instructions
VIP	The level of infection among key figures in the country, including leaders, medical personnel, and security personnel	-1 = New infections
		0 = Unchanged

Dealing	Positive measures taken in order to deal with COVID-19 including publishing economic measures, developing vaccines, testing experimental treatments, and increasing the number of tests available to citizens	1 = Announcement of a positive step
		0 = Otherwise
Education	Level of restrictions in the education system	-1 = New restriction applies
		0 = Unchanged
		1 = Restrictions are removed
Working	Restrictions in workplaces	-1 = New restriction applies
		0 = Unchanged
		1 = Restrictions are removed
Panel B: Calculated metrics		
Ratios	Details	Values
DPI	Deaths per infection—ratio between the number of people death from COVID-19 to the number been infected	Calculated value
RPI	Recoveries per infection—ratio between the number of recoveries and the total number of infections	Calculated value
TPI	Tests per infection—ratio between the number of tests performed and the number of infections	Calculated value
TPD	Tests per death—the ratio of the number of tests performed to the total number of deaths from COVID-19	Calculated value

Note: Table 1 provides a comprehensive description of the study’s variables. In Panel A, the variables are categorized into two primary groups. The first, pandemic impact metrics, consists of four quantitative variables: infections, deaths, recoveries, and tests conducted. The second group, pandemic response metrics, consists of six categorical variables representing governmental and public responses to the epidemic in the form of restrictions, public behavior, VIP, Dealing, education, and working arrangements. Panel B offers a detailed explanation of the four calculated metrics: the ratios of DPI, RPI, TPI, and TPD.

### Methods

To evaluate the impact of COVID-19 on stock markets in the specified country categories, we conducted two distinct regression analyses. The initial regression was tailored for the primary examination. In this regression, the ten collected variables were assigned as independent variables, with stock index returns designated as the dependent variable. In the subsequent robustness analysis, we employed four calculated metrics as independent variables, keeping stock index returns as the dependent variable. The standard regression equation was formulated as follows:

$$AR_{FC,t}=\alpha_{a}+\sum_{i=1}^{I}\beta_{i}X_{a,t}^{i}+\sum_{j=1}^{J}\gamma_{j}C_{a,t}^{j}+\varepsilon$$ (1)

and

$$AR_{MC,t}=\alpha_{a}+\sum_{i=1}^{I}\beta_{i}X_{a,t}^{i}+\sum_{j=1}^{J}\gamma_{j}C_{a,t}^{j}+\varepsilon$$ (2)

*AR*_*FC*,*t*_ represents the average return in countries experiencing low infection rates, while *AR*_*MC*,*t*_ signifies the average return in countries experiencing high infection rates on a given day, denoted as day *t*. Parameter *α*_*a*_ represents a constant term within the regression model. *X*^*i*^ collectively refers to the pandemic impact metric variables, encompassing infections, deaths, recoveries, and tests. *C*^j^ represents the variables associated with government actions and policies, which include restrictions, public behavior, VIP, dealing, education, and working arrangements.

The regression analyses for assessing robustness were as follows:

$$AR_{FC,t}=\alpha_{a}+\sum_{k=1}^{K}\delta_{i}Y_{a,t}^{k}+\varepsilon$$ (3)

and

$$AR_{MC,t}=\alpha_{a}+\sum_{k=1}^{K}\delta_{i}Y_{a,t}^{k}+\varepsilon$$ (4)

*Y*^*k*^ represents the calculated metrics, specifically, DPI, RPI, TPI, and TPD.

## Empirical results

### Descriptive statistics

This section provides an overview of the data through descriptive statistics. Table 2 presents a statistical analysis of the stock indices in the two groups of countries based on the number of infection rates, as gathered from Investing.com. These indices serve as the dependent variables in the regression analysis.

**Table 2:** Descriptive statistics of the research indices.

Country	Index	N	Mean (%)	Median (%)	Std. Dev. (%)	Max (%)	Min (%)
Panel A: Low infection							
Israel	TLV 35	118	-0.173	-0.079	2.158	7.099	-6.699
Austria	ATX	125	-0.235	-0.310	2.952	10.740	-13.650
Slovenia	SBITOP	123	-0.054	0.100	1.758	6.140	-8.960
Argentina	Argentina General	119	-0.004	-0.160	3.974	10.460	-14.850
China	SSEC	117	-0.009	0.110	1.362	3.150	-7.720
Taiwan	TPEX 50	116	0.059	0.305	2.194	7.490	-8.290
Singapore	FTSE Singapore	129	-0.149	-0.090	1.974	6.940	-7.210
New Zealand	NZX 50	125	-0.025	0.000	1.972	14.200	-6.160
Panel B: High infection							
UK	FTSE 100	125	-0.133	0.110	2.345	9.530	-10.820
Spain	Madrid 35	126	-0.185	0.020	2.605	7.820	-14.060
Italy	Milano 40	126	-0.111	0.195	2.850	9.060	-16.640
Sweden	Stockholm 30	123	-0.024	0.130	2.302	7.090	-10.570
France	CAC 40	126	-0.119	0.085	2.563	8.390	-12.280
Germany	DAX	125	-0.025	0.070	2.620	10.980	-12.240
US	S&P 500	125	0.009	0.190	2.893	9.380	-11.980
Brazil	Brazil INDEX 50	123	-0.084	-0.050	3.824	14.700	-14.990

Note: Table 2 provides descriptive statistics for the primary stock indices under examination, categorized into two groups. Panel A displays the findings for countries characterized by low rates of infection, while Panel B presents the results for countries characterized by high rates of infection. The statistical measures (mean, standard deviation, minimum, median, and maximum values) are expressed as percentages.

Table 2 illustrates notable trends in daily return rates across countries in the context of infection rates. Among the countries characterized by a low infection count, Taiwan saw the highest average daily return (0.059%); among the high-infection count group, the United States registered the highest daily return (0.009%). Austria, a country with a relatively low infection rate, reported the lowest daily return (-0.235%) in its group. Spain, a country with a high infection rate, recorded the lowest daily return (-0.185%) in its group. Argentina and Brazil demonstrated considerable index variation, whereas China displayed the most stable index. On average, countries with a low infection rate exhibited a daily return of -0.074%, which surpassed the daily returns of -0.084% among countries with a high infection rate.

Table 3 presents a comprehensive dataset characterizing the countries in our sample with respect to several key COVID-19 indicators, namely the number of infections, deaths, recoveries, and tests conducted, standardized per million residents to facilitate cross-country comparability. The countries featured in Table 3 have been segregated into two panels: Panel A consists of eight countries with relatively low infection rates and Panel B consists of eight countries with higher infection rates. Within these panels, cumulative values were computed for each variable. To ascertain the significance of disparities between the two groups, an independent t-test was conducted to determine the extent to which countries with varying infection rates differed in these critical COVID-19 metrics.

**Table 3:** Cumulative values of stock indices and pandemic impact metric variables.

Panel A: Low infection									
	Israel	Austria	Slovenia	Argentina	China	Taiwan	Singapore^b^	New Zealand	ALL
Return	-20.75%	-30.43%	-8.72%	-8.36%	-3.26%	2.45%	-12.50%	-5.64%	-9.42%**
Infections	2,823.74	1,961.49	762.41	1,377.45	58.9	18.77	7,462.98	244.29	1636.53***
Death	36.86	78.06	53.39	28.32	3.22	0.29	4.44	4.56	23.27***
Recovered	2003.46	1829.59	665.73	487.39	55.33	18.35	6580.81	240.74	1,329.64***
Tests	115,648.60	68,035.60	48,933.30	7,797.23	0^a^	3,228.05	0^a^	83,363.81	47,176.38***
Panel B: High infection									
	UK	Spain	Italy	Sweden	France	Germany	US	Brazil	ALL
Return	-18.69%	-25.38%	-18.67%	-7.98%	-18.30%	-8.03%	-4.84%	-19.80%	-15.21%
Infections	4,595.42	5,331.46	3,976.66	6,700.19	2,516.49	2,318.57	7,826.38	6,436.77	4,962.74
Death	641.89	606.46	574.64	525.78	456.74	107.1	381.09	274.34	446
Recovered	0^a^	3,216.30	3,146.60	0^a^	1,098.67	2,125.70	2,177.12	3,708.69	2,578.84
Tests	87,243.10	74,219.80	89,148.90	0^a^	14,375.51	0^a^	97,578.04	6,956.51	61,586.98

Note: Table 3 shows the cumulative values of the stock indices and pandemic impact metric variables, categorized into two groups. Panel A presents the outcomes for countries characterized by low rates of infection, and Panel B showcases the results for countries characterized by high rates of infection. Statistical significance is denoted by p-values, with asterisks ***, **, and * representing significance at the 1%, 5%, and 10% levels, respectively.

^a^. *Data omissions*: Missing data indicate instances where data points are not available.

^b^. *Singapore inclusion*: Singapore’s inclusion in Panel A is due to its limited susceptibility to the COVID-19 pandemic. Nonetheless, it should be noted that despite its modest size, Singapore experienced a relatively high infection rate in terms of infections per million individuals.

Of the countries characterized by lower infection rates (Panel A of Table 3), Austria and Israel displayed the most significant declines in their respective index returns, with reductions of 30.43% and 20.75%, respectively. Of the countries with higher infection rates (Panel B of Table 3), a specific cluster of five countries experienced substantial declines in their index values: the United Kingdom (-18.69%), Spain (-25.38%), Italy (-18.67%), France (-18.30%), and Brazil (-19.8%). It should be noted that five of the seven indexes exhibiting declines are those of European countries.

Comparison of the panels reveals a substantial divergence in the average cumulative number of infections per million individuals, with Panel A countries reporting a significantly lower average (1,636.53) than Panel B countries (4,962.74). Specifically, Taiwan (18.77) and China (58.9) reported the lowest infection rates, while the United States (7,926.38), Brazil (6,436.77), and Sweden (6,700.19) registered the highest infection rates. In terms of COVID-19-related fatalities, average numbers of deaths per million residents in Panel A countries (23.27) were notably lower than for countries in Panel B (446). The countries with the lowest numbers of deaths were Taiwan (0.29) and China (3.22), while the United Kingdom (641.89), Spain (606.46), and Italy (574.64) reported the highest numbers of deaths.

In terms of recovery from COVID-19, Panel A countries exhibited significantly lower numbers of recoveries per million residents (averaging 1,329.64) than Panel B countries (averaging 2,578.84). Notably, Brazil (3,708.69), Spain (3,216.3), and Italy (3,146.6) reported the highest numbers of recoveries, whereas Taiwan (18.35) and China (55.53) exhibited the lowest figures. Regarding testing efforts, the average numbers of tests conducted per million people were significantly higher in Panel B countries (61,586.98) than in Panel A countries (47,176.38). Israel (115,648.56) and the United States (97,578.04) had the highest testing rates, while Taiwan (3,228.0) and Brazil (6,956.51) reported the lowest numbers of tests administered per million individuals.

**Fig 1:**
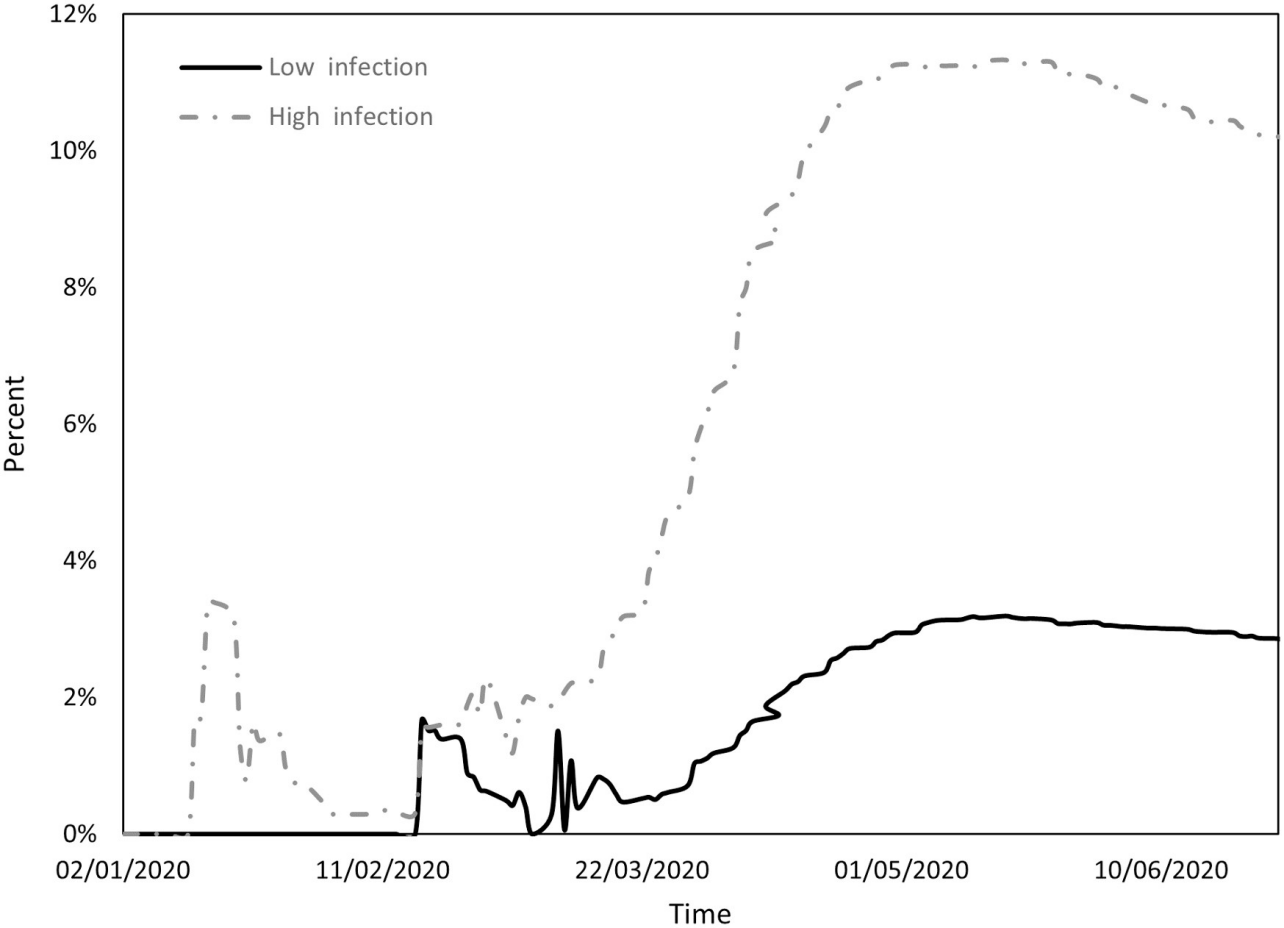
Performance of the DPI index.

**Fig 2:**
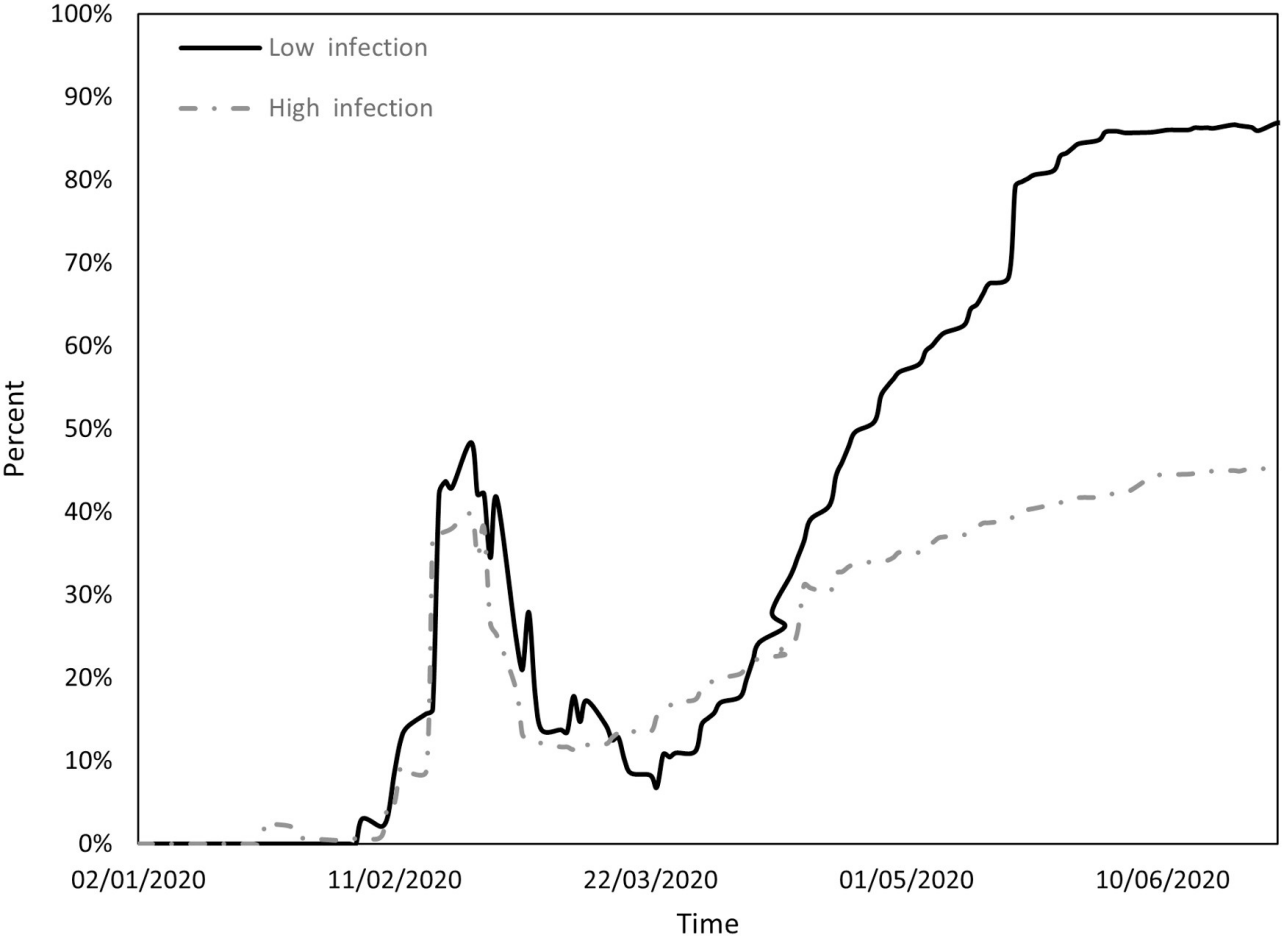
Performance of the RPI index.

**Fig 3:**
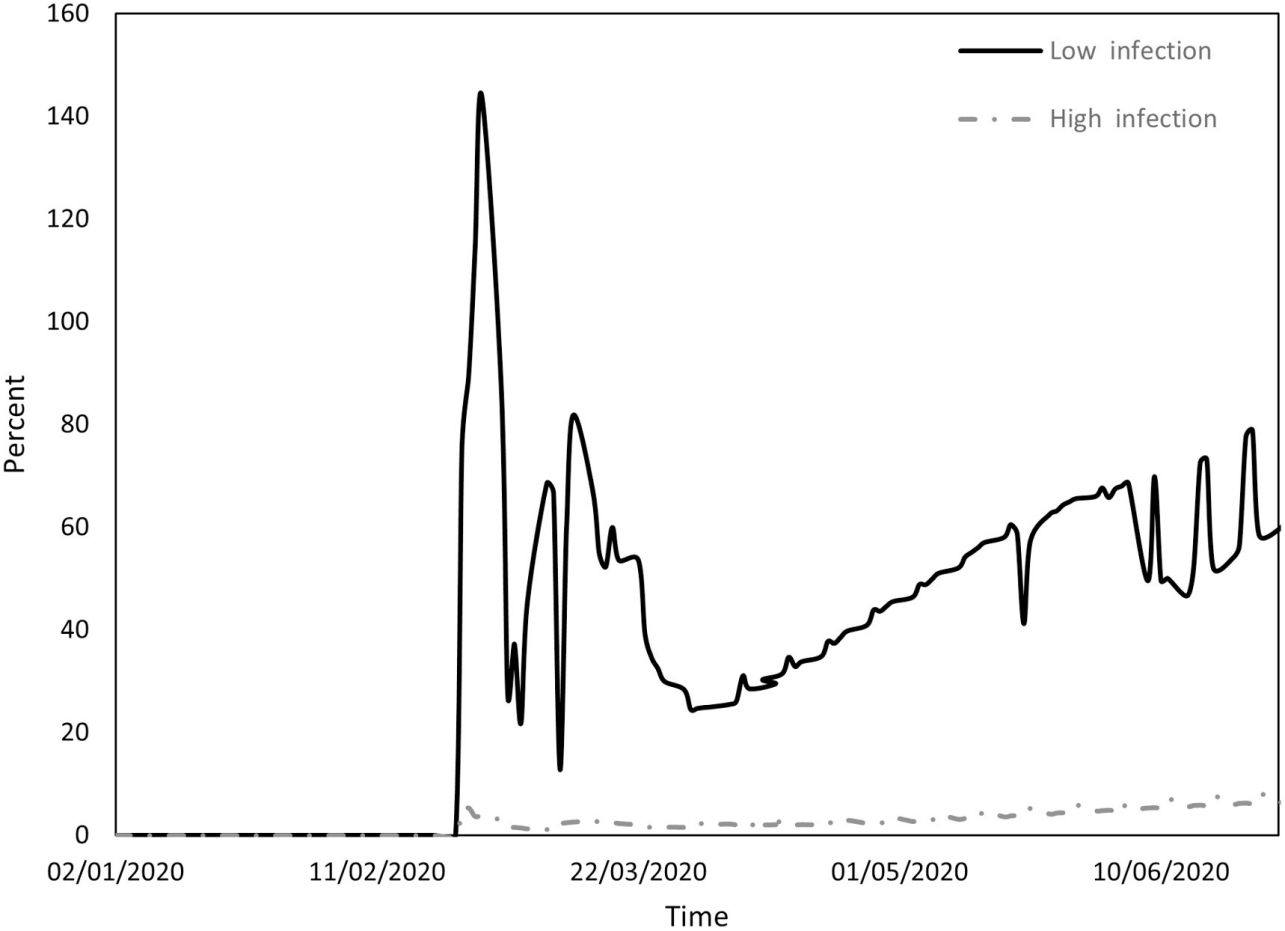
Performance of the TPI index.

**Fig 4:**
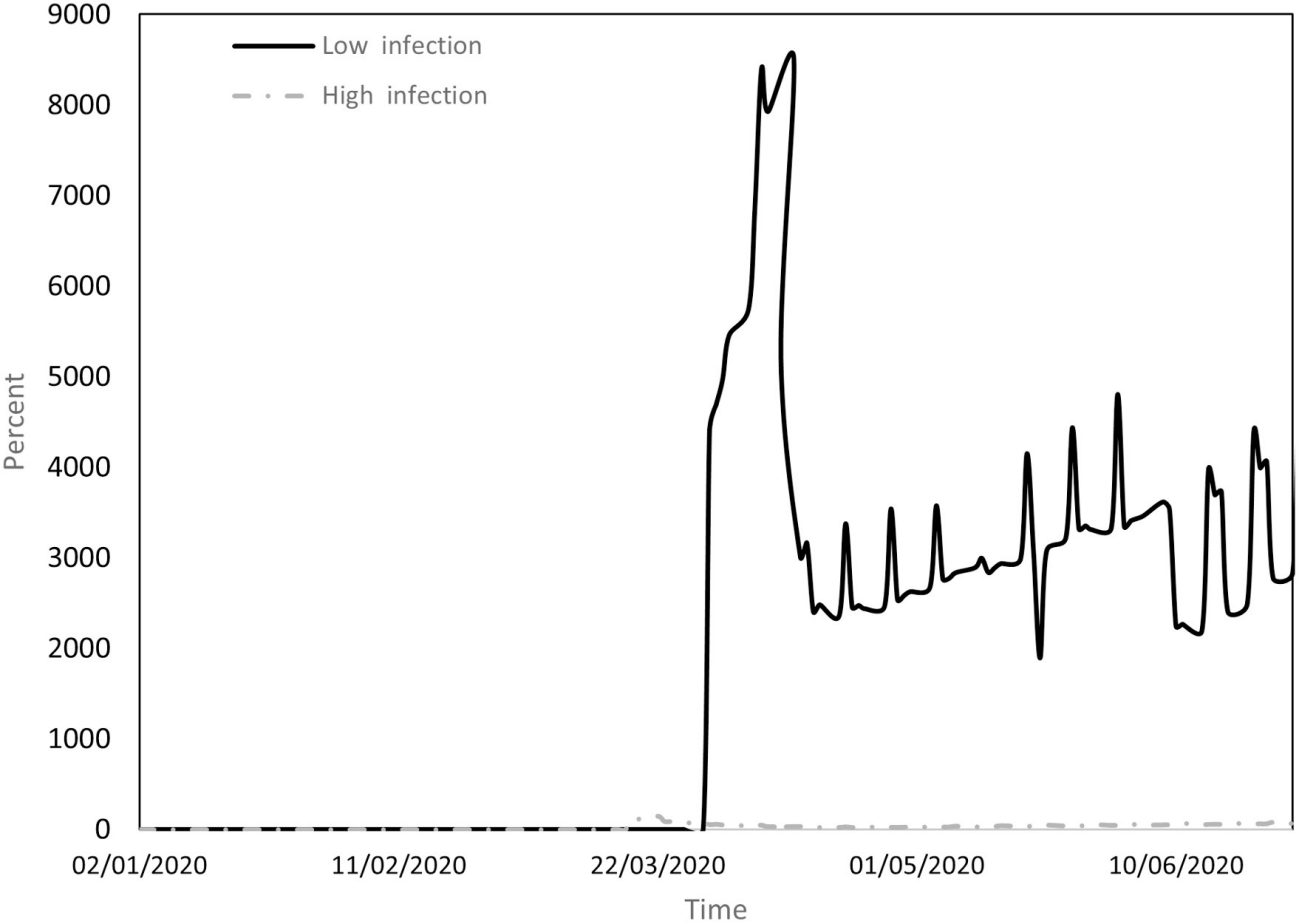
Performance of the TPD index.

Figs 1 to 4 indicate the performance of the indices over the course of the examination period for both cohorts, Panel A countries (depicted by a continuous line) exhibit a low incidence of infections, while Panel B countries (depicted by a dashed line) display a high prevalence of infections. Specifically, Fig 1 shows the ratio between the count of COVID-19 deaths and the count of infections (DPI), Fig 2 the ratio between the counts of recoveries and infections (RPI), Fig 3 the ratio between the counts of tests administered and infections (TPI), and Fig 4 the ratio between the counts of tests administered and deaths (TPD).

Fig 1 reveals three distinct phases within the study period. The initial phase extended until mid-March and was followed by a second phase from mid-March to the end of April and a final phase from the beginning of May to the end of June. During the initial phase, both groups had relatively low DPI values but Panel B countries experienced notably higher values. This phase saw a significant surge in fatalities in Asian countries during January, coinciding with a substantial number of infections. This initial wave of fatalities gradually receded, leading to a decline in DPI that lasted until mid-March. In the second phase, a conspicuous disparity emerged between the groups: Panel B countries experienced an exponential escalation in DPI, whereas Panel A countries observed a more moderate increase. The third phase saw a decline in DPI for both groups, possibly due to the implementation of various restrictive measures by the respective countries.

Fig 2 illustrates that during the initial phase there was no discernible difference between the groups in terms of RPI, which experienced a pronounced increase until mid-February, followed by a decline (primarily affecting Asian countries). In the subsequent phases of transmission and containment, there was a substantial surge in RPI for countries with lower infection counts (Panel A), juxtaposed with a more modest increase for countries with higher infection counts (Panel B). This phenomenon may be attributed to the rapid surge in infections in the former group compared to the gradual increase in the latter.

Fig 3 compares the number of tests conducted and the number of infections (starting from February 24, when data on numbers of tests began to be documented). TPI displayed an upward trend in both cohorts, but with significantly higher values in Panel A countries compared to Panel B countries. A plausible rationale may be found in Table 3, where it is evident that although the number of tests in countries with higher infection counts increased somewhat more than in countries with lower infection counts, the sheer volume of infections in the former group led to a more substantial increase in TPI.

Fig 4 compares the number of tests conducted and the number of deaths (again, starting from February 24, when data on numbers of tests began to be documented). TPD displayed an upward trend in both cohorts, but with significantly higher values in Panel A countries compared to Panel B countries. A plausible rationale may be found in Table 3, where it is evident that, although the number of tests in countries with higher infection counts increased somewhat more than in countries with lower infection counts, the sheer volume of deaths in the former group led to a more substantial increase in TPD.

**Fig 5:**
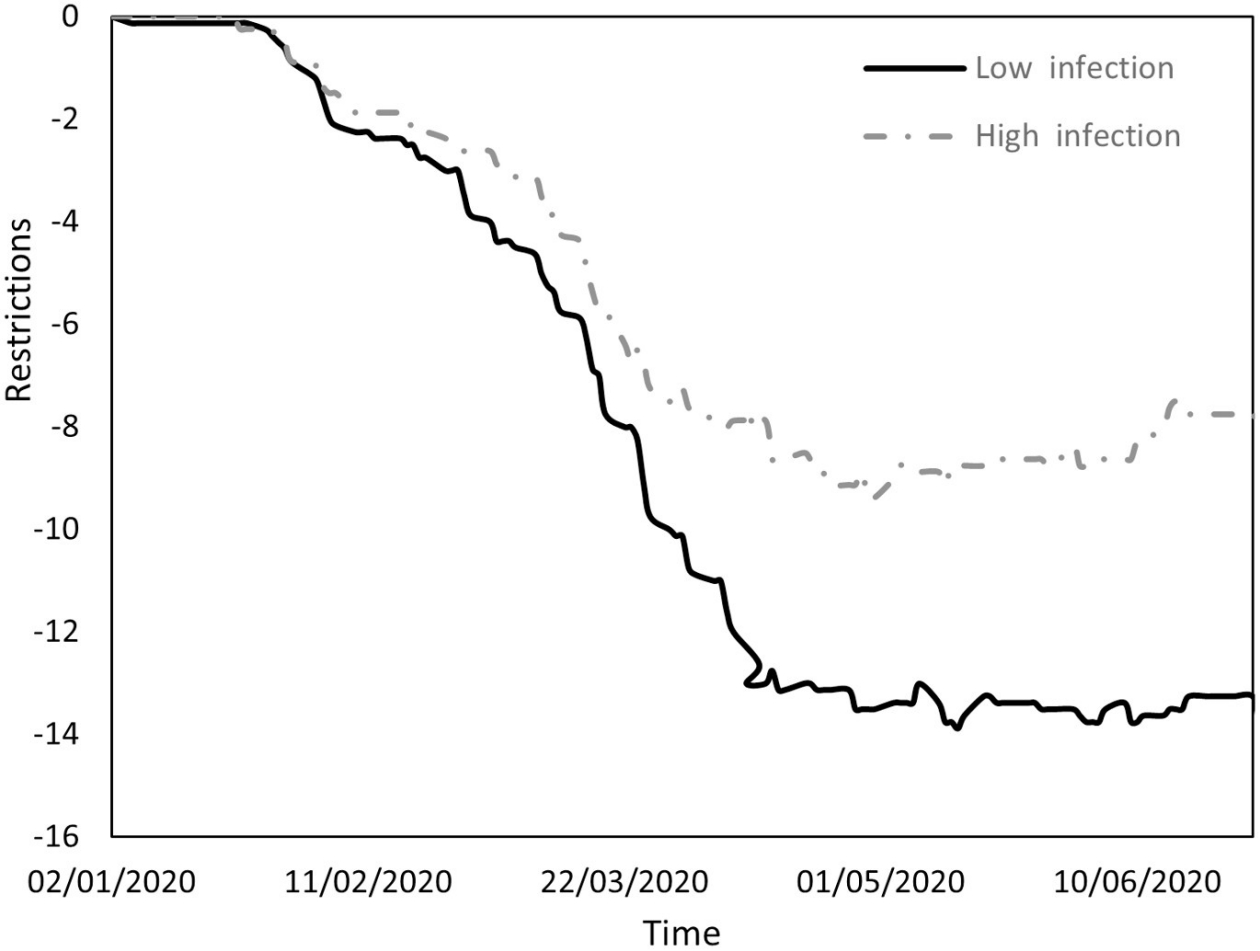
Changes in the restrictions variable.

**Fig 6:**
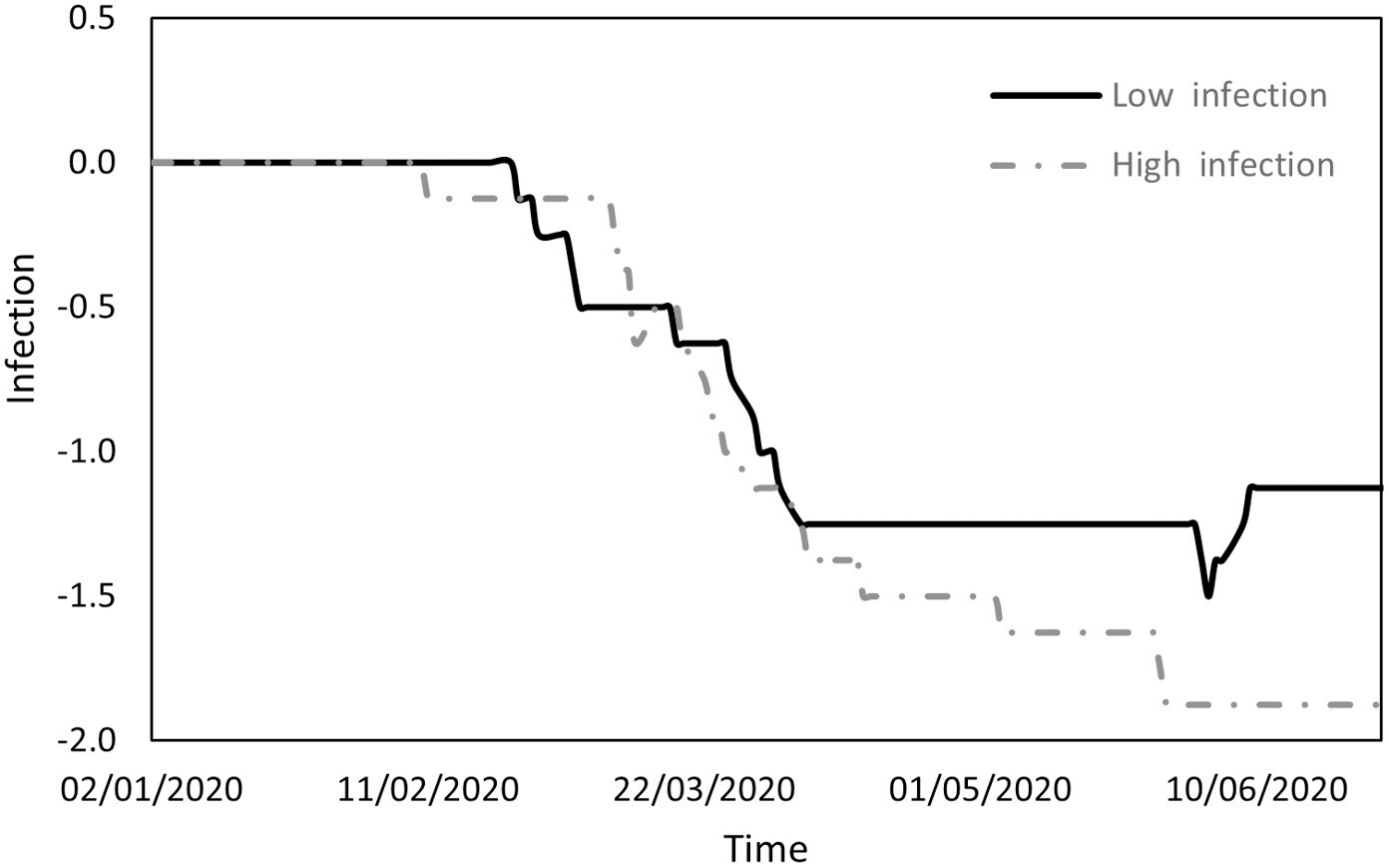
Changes in the VIP variable.

**Fig 7:**
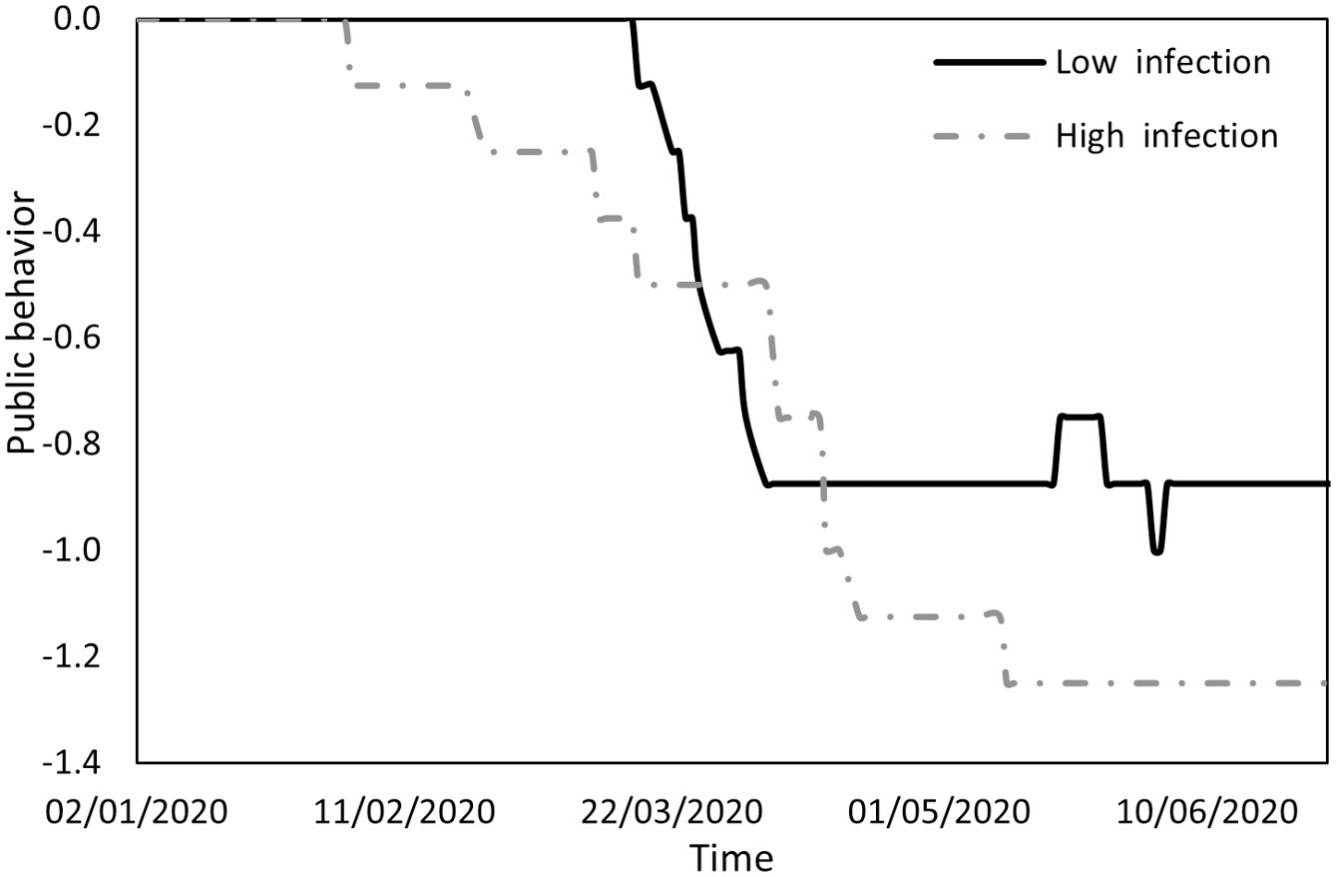
Changes in the Public behavior variable.

**Fig 8:**
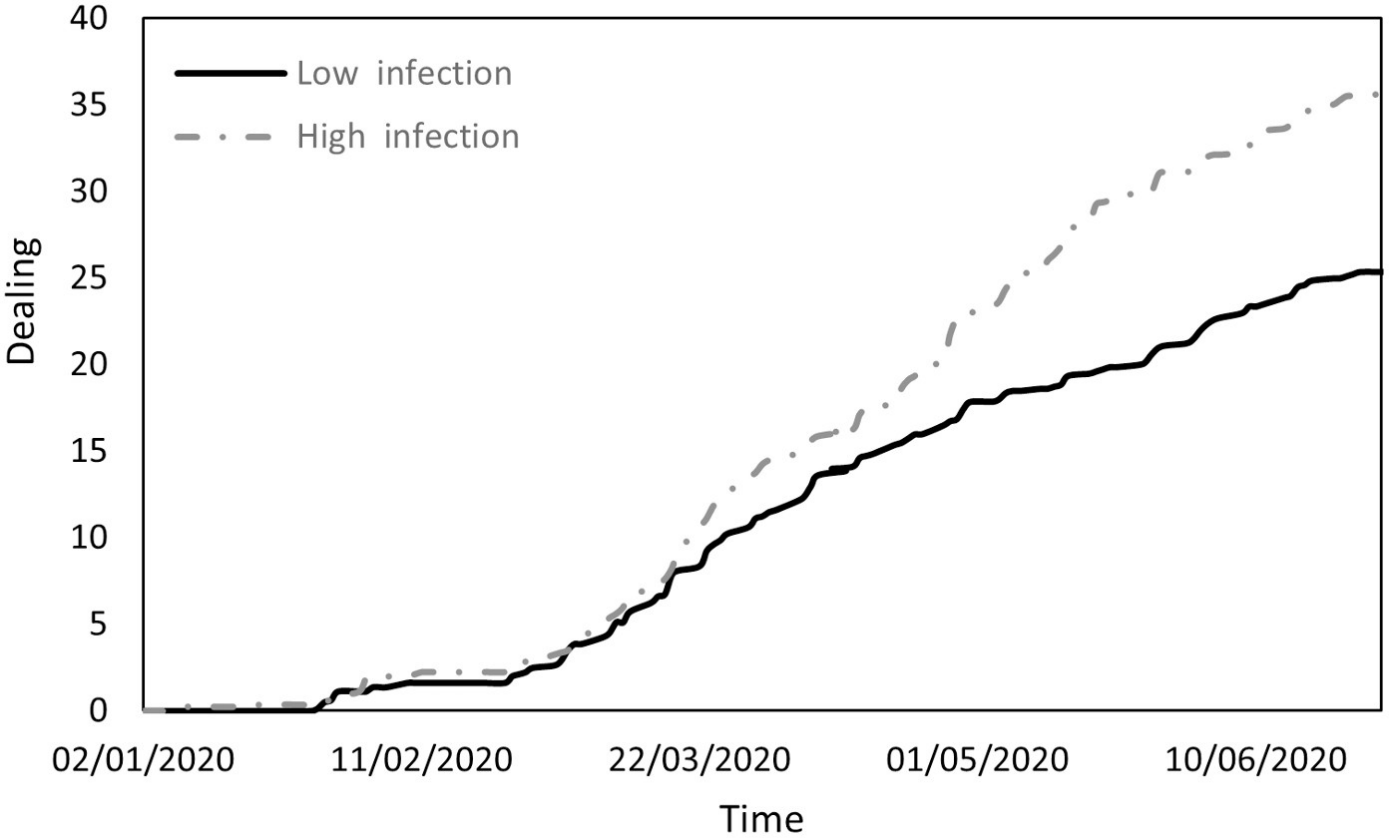
Changes in the dealing variable.

**Fig 9:**
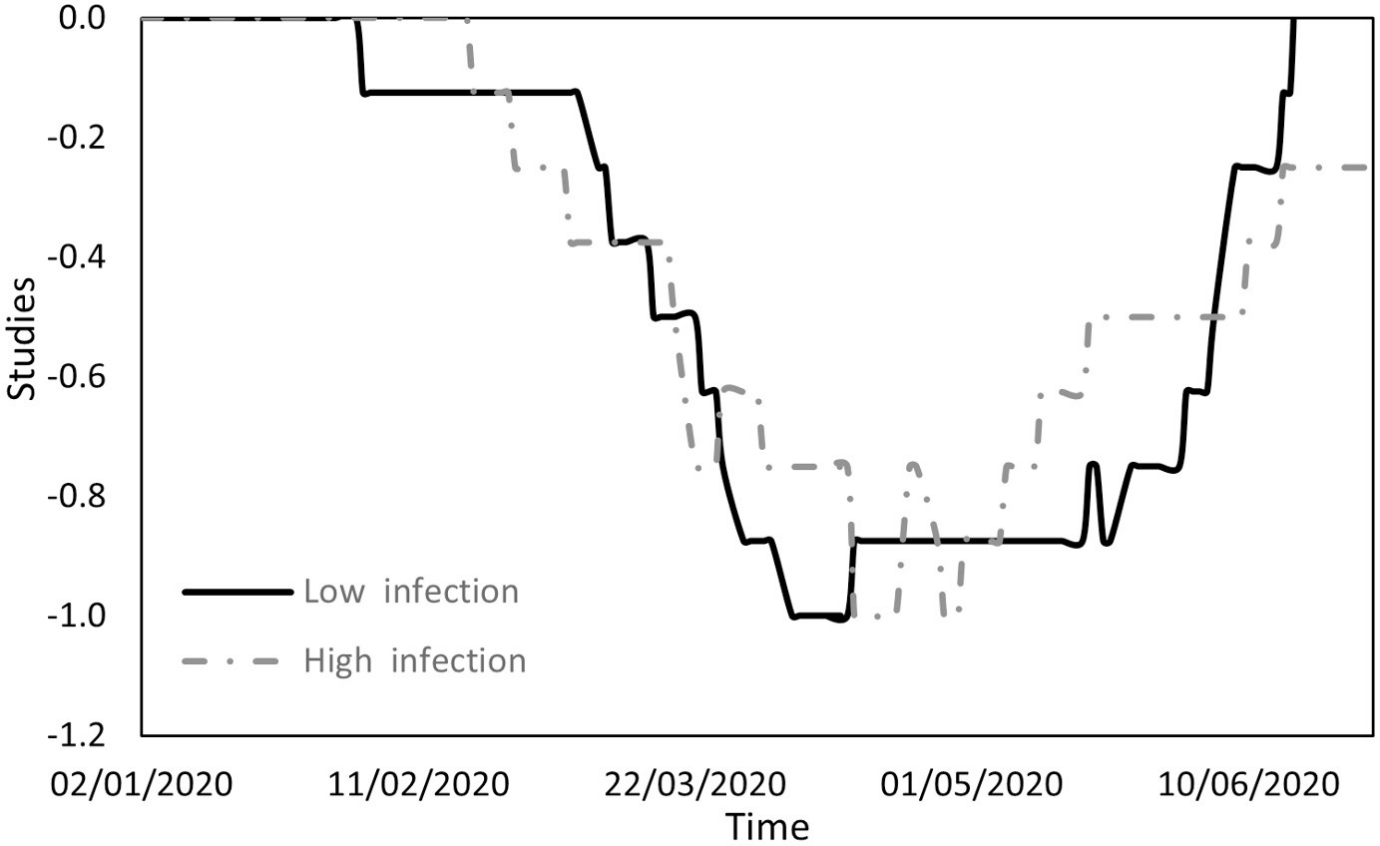
Changes in the education variable.

**Fig 10:**
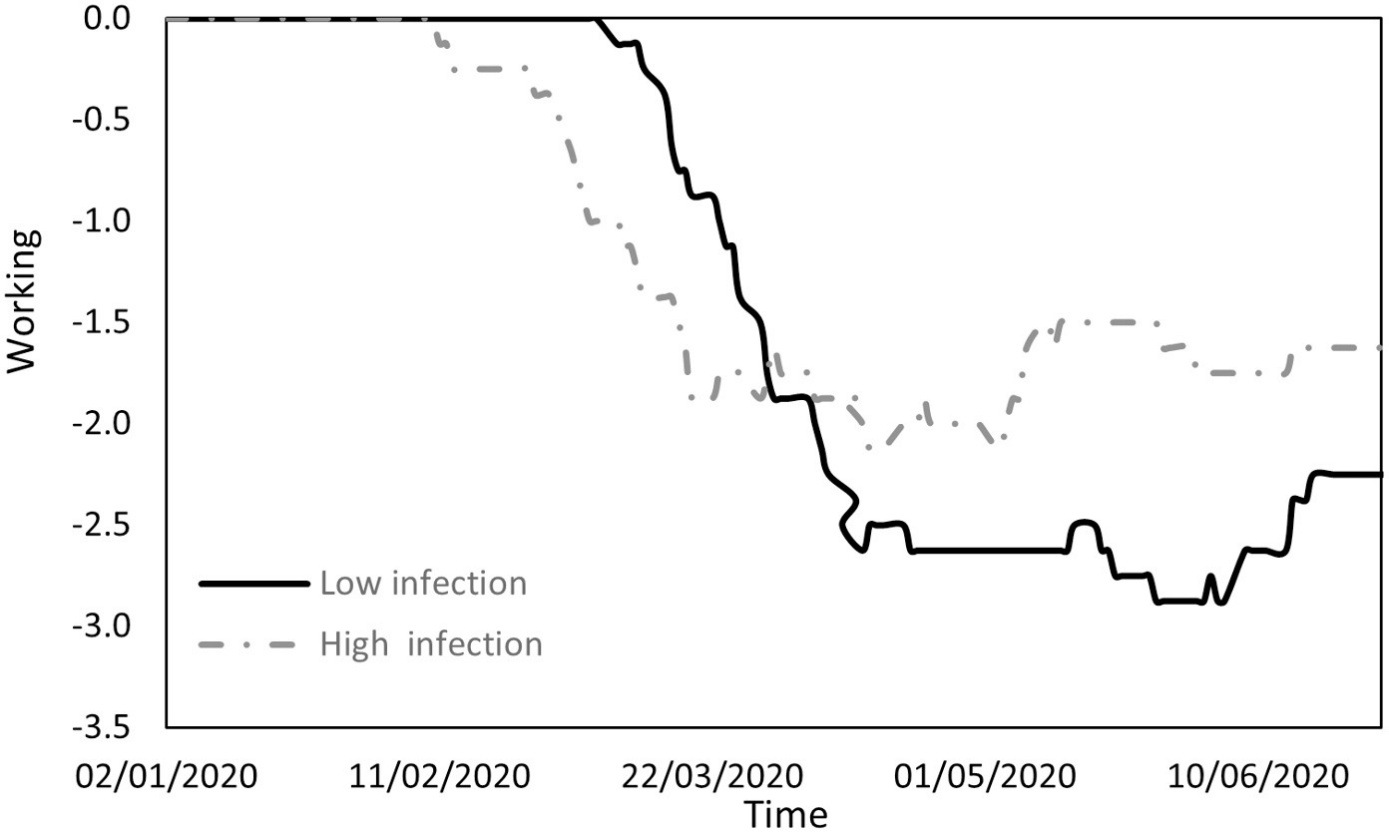
Changes in the working variable.

Figs 5 to 10 provide a comprehensive analysis of the distinct responses and behaviors exhibited by Panel A and Panel B countries during the period under study. Fig 5 offers insight into the average values of an index reflecting a spectrum of restrictions imposed: limitations on movement, lockdowns, quarantine measures, and constraints on incoming international tourists. Until early February, both groups of countries experienced minimal restrictions, resulting in negligible disparities. Subsequently, governments began implementing measures aimed at containing the virus. Panel A countries, with lower infection rates, enforced stringent restrictions, while Panel B countries, marked by higher infection rates, imposed milder restrictions, facilitating transmission of the virus. By mid-April, both groups had reached a state of equilibrium, as indicated by the horizontal lines on the chart.

Fig 6 focuses on the average infection levels among VIP in each group. In both groups of countries, infections were infrequent until mid-February but surged from mid-February to early April. From early April onward, countries with low infection counts reported minimal VIP infections; Panel B countries maintained a moderate, yet persistent trend. This discrepancy may be attributed to the stringent restrictions in countries with lower infection rates, which curbed transmission of the virus to VIP.

Fig 7 shows variations in public responsiveness to government directives. Public behavior oscillated between initial compliance, a subsequent weakening of compliance, and a return to stricter adherence to directives. Extended periods of compliance corresponded to lower infection rates, with compliance eroding to a lesser extent in countries with low infection counts (from mid-March to early April) compared to their high-infection counterparts (from early February to mid-May). These findings, in conjunction with the observations in Fig 6, suggest that, in high-infection countries, responsibility for virus transmission can be attributed to both governments and the general public.

Fig 8 focuses on active measures undertaken by countries to address COVID-19: economic interventions, vaccine development, experimental treatments, and enhanced testing availability for citizens. The trajectory is similar in the two groups. Until the end of February, government involvement remained minimal in both groups. Subsequently, as the impact of the virus on public health and the economy became more apparent, governments-initiated measures to assist their populations. These measures were more robust in countries with higher infection rates than in those with lower infection rates, likely due to the greater health and economic challenges faced by the former group.

Fig 9 describes the educational landscape, which was marked by similar levels of restrictions in both groups and characterized by four discernible phases. Initially, restrictions were virtually absent, with countries with lower infection counts leading slightly in terms of implementing restrictions. Subsequently, concerns about infections in educational settings prompted the gradual imposition of further restrictions. In the third phase, restrictions for both groups were at their height. In the final phase, governments began to ease these restrictions. Although the trends were comparable, countries with lower infection rates experienced a more extended period of maximum restrictions (from mid-April to the end of May) than their counterparts (from mid-April to the end of April), which may have contributed to their lower infection rates.

Fig 10 outlines the work environment, which is also characterized by similarities between the groups and can be divided into three phases. Initially, workplace restrictions were minimal. Subsequently, increasing concern about infection in the workplace prompted governments to introduce restrictions, with Panel B countries initiating these measures three weeks earlier. In the third phase, all countries reached the peak of their workplace restrictions. Countries with lower infection rates enforced more stringent restrictions than those with higher infection rates.

### The impact of model variables on index returns

Applying the ordinary least squares (OLS) regression on systematically collected data, this section investigates the influence of pandemic impact metrics and government actions and policies on the performance of financial indices. Panel A in Table 4 shows the results of the regression analysis for countries characterized by a low incidence of infections, while Panel B present the results for countries marked by a high prevalence of infections.

**Table 4:** Regression estimates of key variables during the pandemic period.

Variable	Panel A: Low infection			Panel B: High infection		
	R^2^ = 0.929, F = 168.731			R^2^ = 0.896, F = 102.085		
	Coefficient	Std. error	t-statistic	Coefficient	Std. error	t-statistic
C	0.018***	0.006	2.924	0.029***	0.008	3.609
Infections	-1.04E-05***	0.000	-2.484	0.000	0.000	-0.136
Death	-2.06E-07***	0.000	-3.438	-1.02E-05***	0.000	-3.125
Recovered	5.91E-05***	0.000	7.148	0.000	0.000	0.216
Tests	5.64E-07***	0.000	2.799	4.26E-08	0.000	1.067
Restrictions	-0.023***	0.005	-4.119	-0.030***	0.012	-2.582
Public behavior	-0.434***	0.063	-6.838	-0.215***	0.076	-2.816
VIP	-0.081***	0.033	-2.451	-0.137***	0.048	-2.878
Dealing	0.048***	0.004	10.789	0.032***	0.006	5.759
Education	0.073**	0.038	1.927	-0.075	0.055	-1.371
Working	0.065***	0.025	2.649	0.144***	0.031	4.607

Note: In Table 4, Panels A and B show the regression results for countries categorized by low and high infection rates, respectively. The set of independent variables comprises pandemic impact metrics and pandemic response metrics. For each panel, the first column gives the regression coefficients, the second column the standard deviations, and the third column the associated t-statistics. Statistical significance is denoted by p-values, with asterisks ***, **, and * representing significance at the 1%, 5%, and 10% levels, respectively.

The regression models are as follows:

$$AR_{FC,t}=\alpha_{a}+\sum_{i=1}^{I}\beta_{i}X_{a,t}^{i}+\sum_{j=1}^{J}\gamma_{j}C_{a,t}^{j}+\varepsilon$$

and

$$AR_{MC,t}=\alpha_{a}+\sum_{i=1}^{I}\beta_{i}X_{a,t}^{i}+\sum_{j=1}^{J}\gamma_{j}C_{a,t}^{j}+\varepsilon$$

In the models, *AR*_*FC*,*t*_ represents the mean return observed in countries with a minimal incidence of infections, while *AR*_*MC*,*t*_ represents the mean return in countries marked by a substantial prevalence of infections at a given time point *t*. Parameter *α*_*a*_ is a constant term. Vector *X*_*i*_ encompasses the variables related to pandemic impact metrics representing infections, deaths, recoveries, and testing. Vector *Cj* encompasses the variables representing government actions and policies, namely restrictions, public behavior, VIP, dealing strategies, education measures, and work-related policies.

The results presented in Table 4 illuminate the impact of the COVID-19 pandemic on index returns in the two groups of countries, those with low infection rates and those with high infection rates. The results reveal that the pandemic-related variables exerted varying degrees of influence on index returns between the groups, thus providing insight into the nuanced dynamics at play. In countries with a low incidence of infections, all the variables under study exhibited statistically significant effects on stock returns. Notably, an increase in the numbers of deaths and infections correlated with a more pronounced decline in the country’s stock market, highlighting the market’s sensitivity to the health and economic risks posed by the pandemic. Conversely, a positive correlation was observed between stock returns and the numbers of recoveries and tests, which is indicative of investor optimism regarding potential recovery scenarios.

Implementation of new government restrictions and a lack of responsiveness from the public were correlated with declines in stock market performance. This relationship suggests that stringent measures and public non-compliance were perceived by the market as detrimental to economic activity, leading to negative market reactions. Conversely, proactive government measures, such as the easing of restrictions in workplaces and educational institutions, contributed to gains in stock market indices. This response may signify the market’s confidence in the potential economic recovery and stability associated with such policy adjustments. In contrast, an increase in the VIP variable, which captures levels of infection among key figures in the country, including political leaders, medical personnel, and security personnel, was associated with decreases in stock returns. This negative correlation can be attributed to concerns over the economic implications of infections within these critical sectors. Infections among key figures may increase uncertainty about the stability and effectiveness of leadership, public health, and security measures, potentially leading to market pessimism.

In countries characterized by a high prevalence of infections, the impact of these variables was comparatively subdued. Although the number of deaths did exhibit a negative effect on stock indices, the numbers of recoveries, infections, and tests did not yield statistically significant effects. The absence of significant effects of these pandemic-related variables in high-infection countries suggests that the market had already integrated the prevailing pandemic conditions into its pricing, thereby reducing the immediate impact of these metrics on stock returns. The negative correlation between government restrictions and public behavior in high-infection countries and stock market performance suggests that the market perceived stringent measures as signaling of prolonged disruption and potential economic strain, and the unfavorable market response may reflect apprehension about the persistence of adverse conditions, given the high infection rates. Conversely, positive market reactions to the easing of workplace restrictions indicate that market participants saw such policy changes as potentially alleviating economic stress and opening up a path to recovery.

The disparities in the influence of pandemic-related variables and government actions between countries with low and high infection rates, respectively, can be attributed to a range of factors. In countries with low infection rates, the heightened sensitivity of stock markets to pandemic metrics such as deaths and infections may reflect market reactions to perceived health and economic risks, with optimism accompanying increases in recoveries and testing. In contrast, the muted market response in high-infection countries could be attributed to ongoing challenges, market adaptation to the persistent pandemic context, and investor skepticism regarding the effectiveness of stringent government measures. These differences underscore the complex interplay of pandemic-related factors and government actions, leading to divergent market responses based on the specific circumstances in each category of countries.

The results in this section support Hypothesis 1, indicating that pandemic impact metrics have different effects on stock indices in countries with different infection rates. This is consistent with research by Tavor and Teitler-Regev [3], who have shown that the type and severity of events have varying impacts on financial markets, underlining the need to consider multiple metrics to understand the specific consequences of health crises. The results in the present study are also in line with the conclusion of Chopra and Mehta [5] those different crises, including the COVID-19 pandemic, have had varying degrees of impact on Asian stock markets, highlighting the non-uniform nature of market responses to such events. In countries with low infection rates, we observed heightened market sensitivity to the pandemic metrics of deaths and infections, with optimism associated with recoveries and testing. In contrast, the impact of these metrics in high-infection countries was subdued, most likely because the markets had already factored the pandemic’s effects into pricing. These findings meet our study’s objective of investigating the impact of COVID-19 on capital markets in 16 countries categorized by infection rates. The divergent market responses underscore the importance of tailoring investment strategies and government policies to country categories. These results have significant implications for policymakers and investors facing the challenges of global health crises, and they provide guidance for navigating financial markets amid extreme uncertainty.

### Robustness checks

The robustness checks conducted in this study used an OLS regression analysis to examine the impact of the DPI (Deaths per infection ratio), RPI (Recoveries per infection ratio), TPI (Tests per infection ratio), and TPD metrics (Tests per death ratio), on index returns. In Table 5, Panel A reports the regression results for countries characterized by a low number of infections, and Panel B reports the results for countries characterized by a high number of infections.

**Table 5:** Regression estimates for calculated metrics during the pandemic period.

Variable	Panel A: Low infection			Panel B: High infection		
	R^2^ = 0.564, F = 47.693			R^2^ = 0.553, F = 34.011		
	Coefficient	Std. error	t-statistic	Coefficient	Std. error	t-statistic
C	-0.049***	0.013	-3.769	-0.051***	0.013	-4.186
DPI	-1.10E+01***	1.48E+00	-7.432	-1.652	1.307	1.264
RPI	0.635***	0.054	11.759	0.219***	0.098	2.237
TPI	2.08E-03***	0.000	8.501	0.005	0.006	0.768
TPD	1.39E-05***	0.000	3.371	0.002***	0.000	5.894

Note: In Table 5, Panels A and B show the regression outcomes for countries categorized by low and high infection rates, respectively. The independent variables are a specific set of calculated epidemiological metrics. Within each panel, the first column presents the regression coefficients, the second column the standard deviations, and the third column the associated t-statistics. Statistical significance is denoted by p-values, with asterisks ***, **, and * representing significance at the 1%, 5%, and 10% levels, respectively.

The regression models are as follows:

$$AR_{FC,t}=\alpha_{a}+\sum_{k=1}^{K}\delta_{i}Y_{a,t}^{k}+\varepsilon$$

and

$$AR_{MC,t}=\alpha_{a}+\sum_{k=1}^{K}\delta_{i}Y_{a,t}^{k}+\varepsilon$$

Where, Y^k^ represents the ratios: *DPI*, *RPI*, *TPI and TPD*.

The results of the robustness analysis lend further support to the findings obtained in the standard tests, reaffirming the substantial influence of the COVID-19 pandemic on stock returns. As detailed in Table 5, even when the analysis uses ratio-based variables, a statistically significant effect of COVID-19 on stock returns is evident for both groups of countries. In countries with a low incidence of infections, a strong association between the examined ratios and stock returns is discerned; specifically, an increase in the ratio of deaths to infections is linked to a corresponding decline in stock returns. Conversely, elevated ratios denoting recoveries per infections (RPI), tests per infections (TPI), and tests per deaths (TPD) are correlated with higher stock returns.

For countries grappling with a high prevalence of infections, the impacts were less pronounced, with only two ratios demonstrating a positive and statistically significant effect on stock returns: increases in the ratio of recoveries per infections (RPI) and in the ratio of tests per deaths (TPD) is were associated with increased stock returns.

In summary, the robustness tests reinforce the conclusions drawn from the standard tests, underscoring the greater sensitivity of stock returns to the examined variables in countries with a low incidence of infections. This phenomenon may be attributed to the proactive policy measures implemented by governments in these countries during the early stages of the pandemic, with each policy adjustment leaving a discernible imprint on stock indices. In contrast, in countries marked by a high number of infections, government interventions occurred at a later juncture, by which time financial markets had already adapted to the ongoing circumstances, leading to a diminished responsiveness of stock indices to changes in the variables under scrutiny.

These findings confirm Hypothesis 2, which predicted varying effects of the calculated metrics on stock indices according to whether countries had low or high infection rates. They also echo insights from the literature review emphasizing the pivotal role that event characteristics and severity play in stock market responses, as noted by Tavor and Teitler-Regev [3] and Chopra and Mehta [5]. The DPI, RPI, TPI, and TPD metrics provide nuanced insights that go beyond basic infection, death, or recovery counts, and they align with the findings of Donadelli et al. [7] and Bai et al. [8] on the influence of information and data on investor attitudes and stock market responses. These metrics constitute vital information for investors when making decisions. The research of Schell et al. [9] highlights the need to consider diverse metrics due to variations in stock market responses to different pandemics. In summary, our results underscore the importance of incorporating calculated metrics such as DPI, RPI, TPI, and TPD when assessing the impacts of a pandemic on financial markets. They carry significant implications for policymakers and investors, emphasizing the need to adapt strategies tailored to the distinct dynamics of stock markets during health crises. These insights provide valuable guidance for navigating financial markets amidst extreme uncertainty.

## Discussion

The year 2020 will be remembered as a year dominated by the unprecedented COVID-19 pandemic. This global crisis has had profound and far-reaching implications, particularly for the world’s economies. Our study delves into the intricate interplay between COVID-19 and capital markets in 16 countries categorized by infection rates. This unique analysis yields a number of valuable insights.

In countries with low infection rates, we observed a heightened sensitivity of stock markets to pandemic metrics. Specifically, stock returns were significantly affected by the numbers of deaths and infections, highlighting the market’s vulnerability to health and economic risks. This sensitivity was mirrored by the positive correlation between stock returns and the number of recoveries and tests, signifying investor optimism regarding potential recovery scenarios. Our findings also emphasize the critical role of government actions and policies in influencing stock market performance. Proactive government measures, such as the easing of restrictions in workplaces and educational institutions, were associated with positive stock market responses. Conversely, stringent measures and public non-compliance were linked to negative market reactions, reflecting the market’s perception of these actions as detrimental to economic activity.

In countries with high infection rates, the impact of pandemic-related variables was notably subdued. Here, the stock market had likely already integrated the ongoing pandemic conditions into its pricing, thereby reducing the immediate influence of these metrics on stock returns. In high-infection countries, the negative correlation between government restrictions and public behavior, on the one hand, and stock market performance, on the other, suggested that the market perceived stringent measures as indicative of prolonged disruption and potential economic strain. Positive market reactions to the easing of workplace restrictions indicated that market participants viewed such policy changes as potentially alleviating economic stress and opening up a path to recovery.

Notwithstanding, it is imperative to recognize specific limitations inherent in our study. A notable limitation is the exclusion of numerous countries affected by COVID-19 from our analysis. Additionally, despite our utilization of data reported by various countries, it is noteworthy that not every country provided comprehensive data, particularly concerning metrics such as the number of tests conducted. Moreover, the diverse measures adopted by different countries introduce potential biases that could impact cross-country comparisons. Nevertheless, the impact of these discrepancies on the results is likely insubstantial. The observed variations in the influence of pandemic-related variables and government actions between countries with low and high infection rates underscore the necessity for bespoke and adaptable strategies in responding to health crises. The distinctive circumstances in each category of countries reflect the intricate interplay between pandemic-related factors and government actions, yielding divergent market responses. However, it is crucial to reiterate the acknowledgment of these identified limitations within our study. The non-inclusion of several COVID-19-affected countries and the variability in data reporting practices among different nations underscore the need for interpretive caution in drawing conclusions from the comparative analyses presented herein.

Future research should incorporate a more extensive array of nations. Moreover, optimal methodological consistency can be achieved by employing a uniform database, if available, that comprehensively encompasses all participating countries. The observed differences in the influence of pandemic-related variables and government actions between countries with low and high infection rates underscore the need for tailored and flexible strategies in response to health crises. The unique circumstances in each category of countries emphasize the complex interplay between pandemic-related factors and government actions, leading to divergent market responses.

## Conclusion and policy implications

### Conclusion

This study illuminates the notable and divergent effects of COVID-19 on financial markets. The pandemic significantly disrupted economies worldwide, and our investigation provides insights into how different infection rates and governmental reactions exerted different influences on stock markets. The heightened responsiveness identified in countries with lower infection rates emphasizes the need for timely and efficient health and economic interventions to mitigate disturbances and uphold market trust. Importantly, these findings offer a nuanced perspective on how governments and policymakers can better respond to similar crises in the future. Although the immediate actions and policies in high-infection countries may not have the same pronounced impact on stock markets, clear and effective communication remains paramount. The ability to maintain public confidence and foster public cooperation is essential in mitigating the economic impact of pandemics. The need for well-calibrated interventions that balance health measures and economic considerations is a critical takeaway for policymakers worldwide.

### Policy implications

The policy implications of our study are both practical and actionable. Policymakers should consider the following three factors.

1. *Flexible policy approaches*: Policymakers should adopt a flexible approach to policy, recognizing that the impact of government actions and policies can vary based on the unique circumstances of each country. This flexibility is essential in responding to the evolving nature of health crises.
2. *Proactive government measures*: Proactive government measures, particularly those that support economic recovery, can have a positive impact on stock market performance. The easing of restrictions in workplaces and educational institutions, when done safely, can be viewed favorably by investors as a potential step toward stability and recovery.
3. *Effective communication*: Effective and transparent communication is crucial. To ensure public understanding and to foster cooperation, policymakers should convey the rationale behind their measures. Public behavior plays a significant role in shaping market reactions, and clarity in communication can help maintain public confidence during health crises.

In conclusion, our study provides a valuable framework for policymakers and market participants to better navigate the complex dynamics of health crises and their impact on capital markets, including practical guidelines for a more effective response to similar challenges in the future.

## Supporting information

S1 File (DOCX)
