# Supplementary material for: Analyzing the varied impact of COVID-19 on stock markets: A comparative study of low- and high-infection-rate countries
Source: PLoS One. 2024 Jan 11;19(1):e0296673. doi: 10.1371/journal.pone.0296673 (PMC10783773; doi:10.1371/journal.pone.0296673)
Supplement: S1 File — (DOCX) [file pone.0296673.s001.docx]

# Appendix A: Data sources

administracion.gob.es. 2020. Retrieved from Nueva normalidad. Crisis sanitaria COVID-19: https://administracion.gob.es/pag_Home/atencionCiudadana/Nueva-normalidad-crisis-sanitaria.html#-bb7ad21ed1cf

Argentina.gob.ar. 2020. Retrieved from nuevo coronavirus COVID-19: https://www.argentina.gob.ar/salud/coronavirus-COVID-19

Bundeskanzleramt. 2020. Retrieved from Bundeskanzleramt - Bundesregierung: https://www.bundeskanzleramt.gv.at/bundeskanzleramt/die-bundesregierung.html

Bundesministerium für Gesundheit. 2020. Retrieved from Coronavirus: https://www.bundesgesundheitsministerium.de/coronavirus/chronik-coronavirus.htmlGouvernement.fr. 2020. Retrieved from Les actions du Gouvernement: https://www.gouvernement.fr/les-actions-du-gouvernement

Congress.gov. 2020. Retrieved from Current Legislative Activities: https://congress.gov/

Gov.il. 2020. Retrieved from News: https://www.gov.il/he/departments/news?limit=10

Gouvernement.fr.2020. Retrieved from Les actions du Gouvernement: https://www.gouvernement.fr/les-actions-du-gouvernement

gov.sg. 2020. Retrieved from Latest update: https://www.gov.sg/features/covid-19

GOV.UK. 2020. Retrieved from Coronavirus (COVID-19) - News and communications: https://www.gov.uk/search/news-and-communications?level_one_taxon=5b7b9532-a775-4bd2-a3aa-6ce380184b6c

Governo do Brasil. 2020. Retrieved from Legislação COVID-19: http://www.planalto.gov.br/CCIVIL_03/Portaria/quadro_portaria.htm

Governo Italiano. 2020. Retrieved from Coronavirus, le misure adottate dal Governo: http://www.governo.it/it/approfondimento/coronavirus/13968

HDX. 2020. Retrieved from Novel Coronavirus (COVID-19) Cases Data: https://data.humdata.org/dataset/novel-coronavirus-2019-ncov-cases

Investing.com. 2020. Retrieved from Investing.com: https://il.investing.com/indices/major-indices

Israel Ministry of Health. 2020. Retrieved from: https://govextra.gov.il/ministry-of-health/corona/corona-virus/?gclid=CjwKCAjw0_T4BRBlEiwAwoEiAaeH0-sYUL95P5WMn0ThqucoCRlSMvw662XArQJRI6OkaaZGK5ZlsRoCNLYQAvD_BwE

New Zealand Legislation. 2020. Retrieved from COVID-19 legislation: http://www.pco.govt.nz/covid-19-legislation/

Regeringen styr Sverige. 2020. Retrieved from Regeringsförklaringen: https://www.regeringen.se/tal/20192/09/regeringsforklaringen-den-10-september-2019/

Republike Slovenije. 2020. Retrieved from Vlada Republike Slovenije: https://www.gov.si/drzavni-organi/vlada/novice/

World Health Organization. 2020. Retrieved from WHO Coronavirus Disease (COVID-19) Dashboard: https://covid19.who.int/

中国政府. 2020. Retrieved from 中国政府: http://www.gov.cn/

台湾政府. 2020. Retrieved from 台湾政府: https://www.president.gov.tw/

| Use | Source |
| --- | --- |
| Spain | Aministracion.gob.es |
| Argentina | Argentina.gob.ar |
| Germany | Bundesministerium für Gesundheit |
| France | Gouvernement.fr |
| Israel | Gov.il |
| UK | GOV.UK |
| Italy | Governo Italiano |
| Brazil | Gov.br |
| Numbers of people who recovered | HDX |
| Stock index | Investing.com |
| Israel | Israel Ministry of Health |
| New Zealand | New Zealand Legislation |
| Singapore | New Zealand Legislation |
| Austria | Österreich |
| Sweden | Regeringen styr Sverige |
| US and VIX | U.S*.*gov |
| Slovenia | Vlada Republike Slovenije |
| COVID-19 data | World Health Organization |
| China | 中国政府 |
| Taiwan | 台湾政府 |

**Appendix B. List of data sources.**

S1: The data used in this research can be found on the figshare depository at 10.6084/m9.figshare.24752037
